# Action Schools! BC: A Socioecological Approach to Modifying Chronic Disease Risk Factors in Elementary School Children

**Published:** 2006-03-15

**Authors:** Patti-Jean Naylor, Heather M Macdonald, Katharine E Reed, Heather A McKay

**Affiliations:** School of Physical Education, Faculty of Education, University of Victoria; School of Human Kinetics, Faculty of Education; School of Human Kinetics, Faculty of Education; Departments of Orthopaedics and Family Practice, Faculty of Medicine, University of British Columbia, Vancouver, British Columbia, Canada

## Abstract

**Background:**

Childhood physical inactivity and obesity are serious public health threats. Socioecological approaches to addressing these threats have been proposed. The school is a critical environment for promoting children's health and provides the opportunity to explore the impact of a socioecological approach.

**Context:**

Thirty percent of children in British Columbia, Canada, are overweight or obese, and 50% of youths are not physically active enough to yield health benefits.

**Methods:**

*Action Schools! BC*, a socioecological model, was developed to create 1) an elementary school environment where students are provided with more opportunities to make healthy choices and 2) a supportive community and provincial environment to facilitate change at the school and individual levels.

**Consequences:**

The environment in British Columbia for school- and provincial-level action on health behaviors improved. Focus group and project tracking results indicated that the *Action Schools! BC *model enhanced the conceptual use of knowledge and was an influencing factor. Political will and public interest were also cited as influential factors.

**Interpretation:**

The *Action Schools! BC *model required substantial and demanding changes in the approach of the researchers, policy makers, and support team toward health promotion. Despite challenges, *Action Schools! BC* provides a good example of how to enhance knowledge exchange and multilevel intersectoral action in chronic disease prevention.

## Background

The prevalence of childhood obesity in Canadian children more than doubled between 1981 and 1996 (1). Similar trends have been identified in other countries and within adult populations. This global pattern is thought to be associated with changes in the social environment, including children's increased exposure to calorie-dense foods and sedentary lifestyle choices and increased barriers to healthy behaviors (2). Chronic diseases in adulthood are potential health consequences of failing to promote physical activity, healthy eating, and healthy weight in children (3).

Ecological models of health promotion are increasingly being promoted (4,5), and researchers have proposed their adoption to combat childhood obesity (2,3). The ecological approach recognizes that human behavior is a consequence of transactions among multiple levels of influence — intrapersonal, interpersonal, organizational or environmental, community, and policy (4,6,7) — and that there is interdependency among levels (5). Socioecological interventions target change strategies at one or more settings or levels directly or indirectly through networking relationships (6).

The school is a critical environment for intervention to promote children's health because the average child spends almost 50% of his or her waking hours in school. Schools also reach children from varied racial and socioeconomic backgrounds (8). Research has demonstrated the potential for setting-based approaches to modify health behaviors such as physical activity (9). However, supporters of the ecological approach suggest that enduring changes in health behavior are best attained through multilevel, multisectoral interventions (4,5). There are currently few models that target multiple levels of influence, including the larger school community (including parents or family), key community partners (e.g., municipal parks and recreation associations), and state-level government (e.g., ministries of health and education) to influence physical activity, healthy eating, and obesity (3).

Forming collaborative partnerships (10) is highly compatible with these multilevel, multisectoral ecological models (5). Partnerships allow for ongoing stakeholder engagement and interaction and have been associated with increased relevance, feasibility, and long-term sustainability of initiatives (10). The importance of these partnerships has been emphasized in both the participatory action research (11) and knowledge exchange literature (12).

*Knowledge exchange* models incorporate the need for a two-way dialogue between researchers and users, incorporate users' needs, and have the potential to increase the impact of knowledge (13,14). The measure of effective knowledge exchange is *knowledge utilization*, which can be *instrumental*, in which a specific research result is the primary influence for a decision, or *conceptual*, in which knowledge has an indirect influence on the thoughts and actions of decision makers (15). Research by Manske (12) on knowledge utilization by public health units highlighted the importance of interactive processes to knowledge utilization in decision making. 

We hypothesized that a multilevel, multisectoral, partnership-based approach would achieve macrolevel changes and influence physical activity promotion in the school setting. For this approach to succeed, multiple stakeholders would guide the development and implementation of a school-level intervention model. We evaluated the efficacy of this model (16) and, using a process evaluation, examined the influence of the ecological partnership-based model on the broader Canadian provincial context (or system)We hypothesized that a multilevel, multisectoral, partnership-based approach would achieve macrolevel changes and influence physical activity promotion in the school setting. For this approach to succeed, multiple stakeholders would guide the development and implementation of a school-level intervention model. We evaluated the efficacy of this model (16) and, using a process evaluation, examined the influence of the ecological partnership-based model on the broader Canadian provincial context (or system). The primary aims of this report are to 1) describe the process of developing and implementing the ecological approach and school-level model and 2) explore the impact of the model at the macro level (provincial environment).

## Context

Among children aged 5 to 17 years in British Columbia (BC), 30% are overweight or obese (17), and 50% of youths aged 12 to 19 years are not physically active enough to achieve health benefits from the activity (18). These estimates, based on telephone surveys, are likely to increase when height, weight, and physical activity are measured directly. In BC, public health agencies have identified physical inactivity and obesity as public health priorities and the school as a priority setting for the primary prevention of chronic diseases (19).

In BC, only 25% of elementary schools devoted the recommended 10% of curriculum time to physical education (PE) in 2001 (20). On average, 80 minutes per week of PE was offered, but approximately 30 minutes of that time was devoted to class management (20). Only three BC school districts employed PE specialists (20). In 2003, education stakeholders opposed a proposed provincial policy requiring mandatory daily PE from grades kindergarten through 12. Within the existing curriculum-based model, school-based physical activity was unlikely to reach the recommended 150 minutes per week.

*Action Schools! BC* (*AS! BC*) was developed in a political environment that valued evidence-based strategies and where the promotion of childhood physical activity was included in the agendas of three government ministries. The BC Ministry of Education was responsible for setting the curricular standards for physical activity, which is mandatory for all students from kindergarten through grade 10 with a recommended allocation of 10% of instructional time. The BC Ministry of Health was responsible for population health and aimed to intensify efforts to promote physical activity, healthy eating, and wellness. The BC Ministry of Tourism, Sport, and the Arts (MTSA) was primarily responsible for physical activity and sport, and its minister publicly committed to increasing physical activity levels in BC to support the Vancouver–Whistler Games. Both the BC Ministry of Health and the Sport Branch of MTSA undertook consultations during 2001 and 2002. They convened key public health, recreation, and sport stakeholders to identify the strategic agenda for action on physical activity. Using schools as a setting for action was a priority. Partners that were currently implementing school-based physical activity initiatives (e.g., JWSporta) and community groups that were championing these initiatives or researching their impact (e.g., BC Recreation and Parks Association, the Heart and Stroke Foundation of BC and Yukon, the University of BC) met to review the evidence and draft a physical activity model.

## Methods

### Engaging partners: the *AS! BC* model

**Figure 1:**
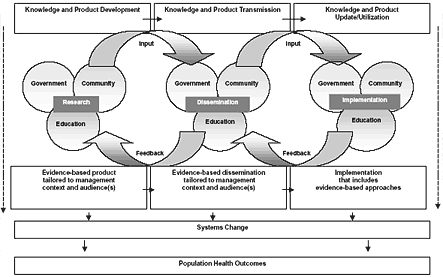
Provincial intervention model for the pilot phase of *Action Schools! BC*, British Columbia, Canada, January 2003 through June 2004. Adapted from Dubois et al (22).

To develop the *AS! BC* model at the provincial level, we reviewed current literature on dissemination of innovations and health promotion. The dissemination literature emphasizes the role of two-way knowledge exchange in the uptake and use of innovations (14,21). The health promotion literature emphasizes the importance of mobilizing strategic alliances when a socioecological approach is being adopted (5,6). We integrated these elements into the *AS! BC *model (Figure 1), which promoted collaboration and exchange of knowledge across sectors. Initially, a research partnership was formed with five agencies in BC: the Ministry of Health, the MTSA, the Ministry of Education, 2010 Legacies Now, and the Provincial Health Services Authority.

Following approval of funding, we formed partnerships horizontally across sectors and vertically, from practitioners to decision makers, by convening three committees. First, a provincial advisory committee (PAC) that met four times per year was formed. The PAC included representatives from core community, school, and government stakeholder groups. Second, the *AS! BC* support team was formed. The support team provided content knowledge and practical experience in developing and implementing a provincewide school-level physical skills program. The support team convened a school advisory committee composed of teachers and principals who contributed to all decisions related to development and delivery of the school-level (grades 4–7) model. (More information on the *AS! BC *partnerships is available from www.actionschoolsbc.ca.)

Third, we formed a multidisciplinary evaluation team of investigators who conducted school- and community-based research trials and evaluated primary health outcomes in the following categories: bone, cardiovascular, obesity, psychosocial, academic performance, healthy eating, and physical activity. Its mandate was to guide study design, research methods, and research tools; collect and interpret data; and disseminate results to stakeholders.

To facilitate knowledge exchange across levels, we established two key processes. First, we ensured there was involvement of individuals across the evaluation and support teams and vertical integration of education stakeholders (i.e., teachers, parents, principals, superintendents, and trustees) on the PAC. Second, we incorporated early and continuous reflection on the evidence and emerging data. This was achieved by 1) discussing evaluation plans and research evidence at each meeting, 2) presenting baseline data within 4 months of beginning the evaluation, 3) providing process evaluation data to program developers immediately and to stakeholders after 6 months, 4) remaining engaged with multiple stakeholders (because they were asked to stay involved beyond the pilot), and 5) actively engaging stakeholders in dissemination and planning for sustainability. In addition, the evaluation and support teams established further connections with stakeholder and community partners (e.g., school superintendents, recreation and parks associations, healthy living coalitions, parent advisory councils) through ad hoc and planned meetings and presentations. Interim data were included in these presentations.

### Evaluating implementation and impact of the model

**Figure 2:**
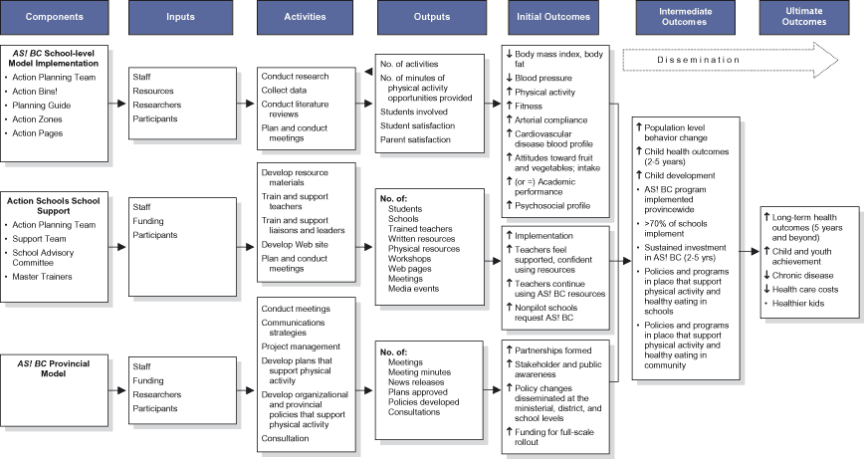
Logic model for pilot phase of *Action Schools! BC *(*AS! BC*), British Columbia, Canada, January 2003 through June 2004.

We used a logic model to guide the evaluation of the *AS! BC* model at the provincial level (Figure 2). We used a descriptive case study design to assess the provincial context for action and the implementation and impact of the *AS! BC* model at the systems level. Focus groups were conducted with the PAC three times over the course of the school-level pilot. Questions were designed to assess the provincial context, including facilitators of, impact of, and barriers to implementation of the model. The support team tracked all formally scheduled meetings with key external stakeholders and community partners in a milestones document that was updated quarterly and circulated to the evaluation team, funders, and the PAC. Media content was collected quarterly using a randomly constructed week methodology (23) to provide an indicator of the public context. Government media releases and funding and policy announcements were tracked as indicators of the political context.

We used an editing analysis approach (24); open coding of the PAC focus group text was conducted using NVivo 7.0 (QSR International, Doncaster, Victoria, Australia). As patterns and themes began to emerge from text units and focus groups, axial coding and clustering were used to generate themes (25). Numbers of text units coded to a theme were counted as an indicator of relative importance. Project meeting tracking records were hand coded, categorized, and counted. We entered reported policy changes and government announcements into a table.

## Consequences

The case study design prevented us from attributing causality to the *AS! BC* model. However, several indicators suggested that the model contributed to changes in the macrolevel context for promoting physical activity and, ultimately, to the sustainability of *AS! BC*.

First, 54% of the PAC focus group passages suggested that *AS! BC* had a positive impact at the macro level. Provincial stakeholders indicated that the *AS! BC *model influenced their strategic approach (evidence of conceptual knowledge utilization). This influence was suggested by the following comments: "We are proposing an Action Women initiative that builds on this but also takes into account the whole challenge of community-level stuff as opposed to school based" and "It is spinning off into our provincial strategy level working with the ministries of health and education."

Other stakeholders emphasized the benefit of the collaboration for developing other provincial initiatives: "It's a marvel [that the] work is interdisciplinary and so interministerial, and we're going to reap the benefit of this with [our initiative]." Stakeholders also indicated that *AS! BC* enhanced political interest: "Action schools kind of galvanized a higher level of interest in several ministries." These shifts in how provincial agencies work enhance the potential for developing ecological solutions to public health problems. 

Second, political announcements provided evidence that the *AS! BC* model was influencing sustainability of physical activity initiatives (instrumental knowledge utilization). For example, on the basis of positive findings from the *AS! BC* evaluation (16), the premier of British Columbia and the ministers of education, health, and finance announced a $14.5 million contribution, over 5 years, for the expansion of *AS! BC*. This directive included 1) implementation of the elementary school model across BC, 2) expansion of the *AS! BC *model for kindergarten through grade 3 and middle school, and 3) development of a secondary school model. An additional $500,000 has been provided by the Ministry of Education for teacher training and to support school districts that enrolled in *AS! BC*. In addition, the following projects were announced: 1) an initiative to promote healthier foods within the school system and eliminate the sale of junk food by 2009, 2) a recognition program for schools to reward health promotion and to promote the spirit of the Vancouver–Whistler Games, 3) development of a provincial framework to promote health through the school setting, 4) delivery of a provincial healthy schools forum, 5) development of new standards for physical education with performance descriptions, and 6) funding support for a Pan-Canadian Consortium for School Health.

Third, during an 18-month period, the project team had 150 meetings with stakeholders (two thirds at the provincial level) that were *not* specifically related to project development and implementation. These additional meetings indicated that the *AS! BC* model provided opportunities for further collaboration on related initiatives and the potential to influence the strategic decisions of stakeholders.

The context surrounding the development and implementation of *AS! BC *may have positively influenced systems-level changes. It was evident from the PAC focus groups that stakeholders viewed the public context as important. The Vancouver–Whistler Games, media and public awareness, renewed interest in public health, leadership, government policies, the need for resources in the school system, and research all emerged as key themes. In addition, media analysis showed a shift in the distribution of the chronic disease prevention discourse from a strong focus on tobacco (1999–2004) to an evenly distributed discourse on tobacco, physical activity, healthy eating, and obesity (23).

## Interpretation

We developed a flexible model to promote physical activity in schools that was 1) based on principles of health promotion and knowledge exchange, 2) involved stakeholders from multiple sectors, and 3) facilitated the development and implementation of plans based on identified needs and priorities. This approach enhances the impact and sustainability of health promotion initiatives (10). Impact at the systems level is measured by changes in public policies or organizational practices including legislation, funding, procedures, regulations, and incentives (26). We observed policy development and changes in funding and regulations that were attributed to, or temporally associated with, implementation of the *AS! BC* model. However, given the context within which *AS! BC* was implemented, it is not possible to attribute these changes definitively to the influence of the model.

Context played a critical role in providing an opportunity to adopt a socioecological approach. The public and political focus on physical activity was increasing in BC, and champions of physical activity were in place and collaborating at many levels. Collaboration requires commitment of resources and political will. Ministers from three government ministries provided resources and participated in the media launch of *AS! BC*, indicating the will to collaborate.

The *AS! BC* model provided an opportunity to adopt a socioecological approach and demonstrated the challenges of doing so. This approach is complex (2) and demands intervention and evaluation across multiple levels and settings. *AS! BC *addressed one setting (the school) with two levels of influence on children's health (local and provincial) within the setting. We did not address broader social and economic policies that are known to affect the health of populations. In addition, although interventions at the systems level have a greater potential for impact, it is more difficult to evaluate their effect using conventional means (26).

The knowledge-exchange–based model required that the evaluation and support teams be extremely responsive and flexible. For example, so that data could be used in decision making, data analysis and reporting timelines were compressed. These teams also exceeded the demands of model delivery and evaluation by providing results and giving public presentations as requested by key stakeholders. They also responded to issues or initiatives that emerged during interactions with stakeholders. Because interaction was a cornerstone of the model, agenda-setting meetings were required, which placed an additional resource demand on the evaluation and support teams.

*AS! BC* illustrates how knowledge utilization is enhanced through multilevel action among sectors and highlights important factors to consider when adopting ecological approaches to chronic disease prevention.

## References

[1] Tremblay M, Willms JD (2000). Secular trends in the body mass index of Canadian children. CMAJ.

[2] Kumanyika S, Jeffery RW, Morabia A, Ritenbaugh C, Antipatis VJ, Public Health Approaches to the Prevention of Obesity (PHAPO) Working Group of the International Obesity Task Force (IOTF) (2002). Obesity prevention: the case for action. Int J Obes Relat Metab Disord.

[3] Raine KD (2004). Overweight and obesity in Canada: a population health perspective.

[4] King AC, Stokols D, Talen E, Brassington GS, Killingsworth R (2002). Theoretical approaches to the promotion of physical activity: forging a transdisciplinary paradigm. Am J Prev Med.

[5] Stokols D (1996). Bridging the theoretical and applied facets of environmental psychology. Am Psychol.

[6] McLeroy KR, Bibeau D, Steckler A, Glanz K (1988). An ecological perspective on health promotion programs. Health Educ Q.

[7] Stokols D (1992). Establishing and maintaining healthy environments. Toward a social ecology of health promotion. Am Psychol.

[8] Fox KR, Cooper A, McKenna J (2004). The school and promotion of children's health-enhancing physical activity: perspectives from the United Kingdom. JTPE.

[9] McKenzie TL, Stone EJ, Feldman HA, Epping JN, Yang M, Strikmiller PK (2001). Effects of the CATCH physical education intervention: teacher type and lesson location. Am J Prev Med.

[10] Gillies P (1998). Effectiveness of alliances and partnerships for health promotion. Health Promot Int.

[11] Israel BA, Schulz AJ, Parker EA, Becker AB, Allen A, Guzman JR, Minkler M, Wallerstein N (2002). Critical issues in developing and following community-based participatory research principles. Community-based participatory research for health.

[12] Manske SR (2001). Explaining knowledge use among clients of the Program Training & Consultation Centre [doctoral thesis].

[13] Lee RG, Garvin T (2003). Moving from information transfer to information exchange in health and health care. Soc Sci Med.

[14] King L, Hawe P, Wise M (1998). Making dissemination a two-way process. Health Promot Int.

[15] Cousins JB, Leithwood KA (1993). Enhancing knowledge utilization as a strategy for school improvement. Knowledge: Creation, Diffusion, and Utilization.

[16] McKay HA, Naylor PJ, Rhodes R, Warburton D, Reed K, Macdonald H (2004). Action Schools! BC phase I (pilot) evaluation report and recommendations.

[17] (2005). National longitudinal survey of children and youth 2002-03 (Cycle 5) [database on the Internet].

[18] Table 105-0233: Leisure-time physical activity, by age group and sex, household population aged 12 and over, Canada, provinces, territories, health regions (June 2003 boundaries) and peer groups, every 2 years [database on the Internet].

[19] Kendall PRW (2003). An ounce of prevention — a public health rationale for the school as a setting for health promotion: a report of the Provincial Health Officer.

[20] Deacon BW (2001). Physical education curriculum review report.

[21] Brown JS, Duguid P (2001). Knowledge and organization: a social-practice perspective. Organization Science.

[22] Dubois N, Wilkerson T, Hall C (2003). A framework for enhancing the dissemination of best practices.

[23] Wharf Higgins J, Naylor PJ, Berry T, O'Connor B, McLean D "The health buck stops where?" Thematic framing of health discourse to understand the context for CVD prevention. J Health Commun.

[24] Crabtree BF, Miller WL (1999). Doing qualitative research.

[25] Miles MB, Huberman AM (1994). Qualitative data analysis: an expanded sourcebook.

[26] Nutbeam D (1998). Evaluating health promotion: progress, problems and solutions. Health Promot Int.

