# Supplementary figures and images for: Action Schools! BC: A Socioecological Approach to Modifying Chronic Disease Risk Factors in Elementary School Children
Source: Prev Chronic Dis. 2006 Mar 15;3(2):A60. (PMC1563946)

**Figure 1.** AS! BC Provincial Intervention Model – Pilot Phase 2003-2004

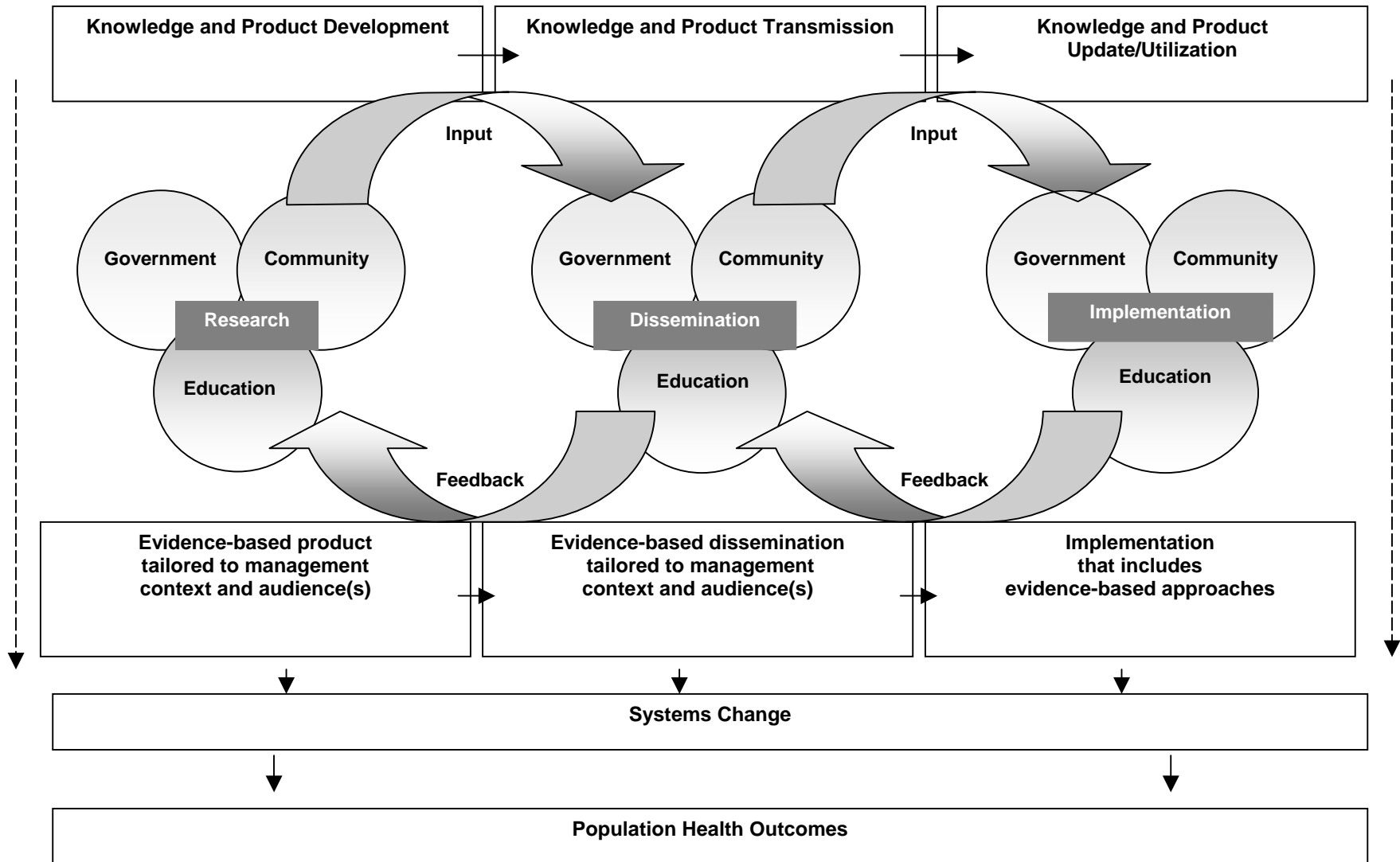

Supplement: Supplementary file 1 [file 05_0090_01.pdf]
